# Proteinase-activated receptor 4 stimulation-induced epithelial-mesenchymal transition in alveolar epithelial cells

**DOI:** 10.1186/1465-9921-8-31

**Published:** 2007-04-16

**Authors:** Seijitsu Ando, Hitomi Otani, Yasuhiro Yagi, Kenzo Kawai, Hiromasa Araki, Shirou Fukuhara, Chiyoko Inagaki

**Affiliations:** 1 Department of Pharmacology, Kansai Medical University, 10-15, Fumizono-Cho, Moriguchi, Osaka 570-8506, Japan; 2 The First Department of Internal Medicine, Kansai Medical University, 10-15, Fumizono-Cho, Moriguchi, Osaka 570-8506, Japan; 3 Fuso Pharmaceutical Industries, Ltd., Joto-ku, Osaka 536-8523, Japan

## Abstract

**Background:**

Proteinase-activated receptors (PARs; PAR_1–4_) that can be activated by serine proteinases such as thrombin and neutrophil catepsin G are known to contribute to the pathogenesis of various pulmonary diseases including fibrosis. Among these PARs, especially PAR_4_, a newly identified subtype, is highly expressed in the lung. Here, we examined whether PAR_4 _stimulation plays a role in the formation of fibrotic response in the lung, through alveolar epithelial-mesenchymal transition (EMT) which contributes to the increase in myofibroblast population.

**Methods:**

EMT was assessed by measuring the changes in each specific cell markers, E-cadherin for epithelial cell, α-smooth muscle actin (α-SMA) for myofibroblast, using primary cultured mouse alveolar epithelial cells and human lung carcinoma-derived alveolar epithelial cell line (A549 cells).

**Results:**

Stimulation of PAR with thrombin (1 U/ml) or a synthetic PAR_4 _agonist peptide (AYPGKF-NH_2_, 100 μM) for 72 h induced morphological changes from cobblestone-like structure to elongated shape in primary cultured alveolar epithelial cells and A549 cells. In immunocytochemical analyses of these cells, such PAR_4 _stimulation decreased E-cadherin-like immunoreactivity and increased α-SMA-like immunoreactivity, as observed with a typical EMT-inducer, tumor growth factor-β (TGF-β). Western blot analyses of PAR_4_-stimulated A549 cells also showed similar changes in expression of these EMT-related marker proteins. Such PAR_4_-mediated changes were attenuated by inhibitors of epidermal growth factor receptor (EGFR) kinase and Src. PAR_4_-mediated morphological changes in primary cultured alveolar epithelial cells were reduced in the presence of these inhibitors. PAR_4 _stimulation increased tyrosine phosphorylated EGFR or tyrosine phosphorylated Src level in A549 cells, and the former response being inhibited by Src inhibitor.

**Conclusion:**

PAR_4 _stimulation of alveolar epithelial cells induced epithelial-mesenchymal transition (EMT) as monitored by cell shapes, and epithelial or myofibroblast marker at least partly through EGFR transactivation via receptor-linked Src activation.

## Background

Proteinase-activated receptors (PARs) are newly identified G-protein-coupled receptors that can be activated by serine proteinases such as thrombin, trypsin, mast cell tryptase and neutrophil cathepsin G [1,2]. These proteinases cleave the extracellular amino terminal domain of PARs to create a new NH_2 _terminal sequence, which functions as a tethered ligand to initiate each receptor-coupled cell signaling. To date, four PARs have been cloned; PAR_1, _PAR_3 _and PAR_4 _are preferentially activated by thrombin, while PAR_2 _are selectively activated by trypsin [2]. In the respiratory system, PAR_1_, PAR_2 _and PAR_4 _are expressed at different levels depending on the tissues or the cell types (epithelium, endothelium, tracheal smooth muscle and blood vessel), and reportedly modulate cytoskeletal structure and further contribute to the progression of various airway and lung disorders including inflammation and fibrosis [2-4]. For example, in systemic sclerosis patients with pulmonary fibrosis or idiopathic pulmonary fibrosis (IPF) patients, concentrations of thrombin and/or cathepsin G in bronchoalveolar lavage fluid are much higher than those in healthy controls [5,6]. Therefore, thrombin receptors such as PAR_1 _and/or PAR_4 _in lung are thought to contribute to the pathogenesis of lung fibrosis. Indeed, Howell et al [3] demonstrated that bleomycin-induced fibrotic responses such as collagen accumulation and increases in profibrotic mediator levels were attenuated by PAR_1_-knockout, suggesting the involvement of PAR_1 _signal in the pathogenic mechanisms. However, contribution of another thrombin receptor, PAR_4_, has not been examined. In our recent study, PAR_4 _(mRNA/protein) has been demonstrated to be highly expressed in primary cultured mouse alveolar epithelial cells [7]. This enabled us to test the involvement of PAR_4 _stimulation in pathogenetic mechanisms of fibrosis in vitro.

Pulmonary fibrosis is a final common endpoint pathomechanism in various lung diseases including acute respiratory distress syndrome (ARDS) [8]. The process is characterized by multiple phenomena such as epithelial activation and damage, an excessive extracellular matrix deposition and a substantial increase in the number of fibroblasts/myofibroblasts [9], transforming growth factor-β (TGF-β), interleukin-4 and tumor necrosis factor-α being known as inducers of such fibrotic responses [8,9]. Recently, phenotypic transition of epithelial cell to mesenchymal cell (epithelial-mesenchymal transition; EMT) has received attention as an important mechanism of progressive increase in the number of myofibroblasts in various fibrotic tissues including kidney and lung [10-12]. Typical alveolar epithelia form a cobblestone-like sheet structure that tightly adhering to neighboring cells or various basal substrates, and play an active role in protecting lung from injury and infection [13]. Under persistent lung pathogenic insults, integrity and characteristics of alveolar epithelium are disturbed and rearranged to induce morphological or physiological alterations, for example, a loss of cell-cell contact, apoptosis and proliferation. Further, parts of epithelial cells are phenotypically changed to different types of cells like mesenchymal cell, i.e., EMT [9,12]. During EMT, the epithelial cells lose their characteristic morphology through a various intermediate stages like a loss of epithelial adhesion molecules such as E-cadherin (a specific epithelial marker) and secretion of matrix metalloproteinase (MMP). Finally, cells are converted to a mesenchymal phenotype and acquire myofibloblast morphology characterized by an elongated cell shape and de novo expression of α-smooth muscle actin (α-SMA, a hallmark of myofibroblast). In several different studies, not only growth factor such as TGF-β, epidermal growth factor (EGF) and interleukin-1 but also some drugs (cyclosporine A and angiotensin ll) have been shown to induce EMT of tubular and alveolar epithelial cells, thereby facilitating the progression of renal and lung fibrosis [8,10,14-16].

In the present study, we examined whether stimulation of PAR_4 _which is known to be involved in the long-scale cellular responses [17] modulates epithelial morphology through EMT using primary cultured mouse alveolar epithelial cells and a human lung carcinoma-derived alveolar epithelial cell line (A549 cells). Possible mechanisms of the PAR_4_'s effects were also analyzed with respect to the involvement of EGF receptor (EGFR) signaling, since this receptor is reportedly transactivated by various extracellular stimuli such as G protein-coupled receptors including PARs [18-20].

## Methods

### Materials

An agonist peptide for PAR_4 _(AYPGKF-NH_2_), and an inactive reverse sequence peptide (FKGPYA-NH_2_) were synthesized by Fuso Pharmaceutical Industries, LTD., Research and Development Center (Osaka, Japan). Thrombin and monoclonal anti-α-smooth muscle actin (α-SMA) antibody (Fluorescein isothiocyanate (FITC) conjugated purified mouse immunoglobulin) were purchased from Sigma chemicals (St. Louis, MO, USA). Joklik's modification Eagle's medium (JMEM), Dulbecco's modification Eagle's media (DMEM) and fetal calf serum (FCS) were from Gibco BRL Life Technologies (Grand Island, NY, USA); human TGF-β1 was from Peprotech EC (Margravine Rode, London, UK); antibodies against E-cadherin, GAPDH and phospho-EGFR (specific for Tyr1173) were from Santa Cruz Biotechnology, Inc., (Santa Cruz, CA, USA); anti-Src and anti-phospho-Src (specific for Tyr416) antibodies were from Cell Signaling Technology, INC (Danvers, MA, USA); 4-Amino-5-(4-chlorophenyl)-7-(t-butyl) pyrazolo [3,4-d pyrimidine (PP2) and 4-(3-Chloroanilino-6,7-dimethoxyquinazoline (AG1478) were from BIOMOL International, L.P., (Matford court, UK); rhodamine red-conjugated goat anti-rabbit IgG was from Jakson Immunoresearch Laboratories, (West Grove, PA, USA); peroxidase-conjugated goat anti-rabbit or anti-mouse immnogloblin was from Cappel, ICN Pharmaceutical, Inc., (Aurora, OH, USA) or Zymed Laboratories Inc., (South San Francisco, CA, USA), respectively; ECL (enhanced chemiluminescence) kit was from NEN Life Science Product (Boston, MA, USA); and SCH-79797 dihydrochloride [(N^3^-Cyclopropyl-7-[[4-(1-methylethyl)phenyl]methyl]-7H-pyrrolo [3,2-f]quinazoline-1,3-diamine dihydrochloride was from Tocris Cookson Ltd. (Bristol, BS11 9XJ, UK). All other chemicals were purchased from Wako Pure Chemical (Osaka, Japan) and of the highest purity available.

### Preparation of primary cultured alveolar epithelial cells

All animals were handled in accordance with the "Rules of Animal Experimentation Committee, Kansai Medical University".

Healthy C57BL/6J mice (20–25 g) were anesthetized with intraperitoneal injection of pentobarbital (1.5 mg/10 g body weight). Primary cell culture was accomplished using protocol described previously with a slight modification [21]. Briefly, the pulmonary vessel was perfused via the right cardiac ventricle with saline. The trachea was cannulated with 3 Fr tubing, and the lung was filled with 1–2 ml of JMEM containing 0.1 % dispase (Godo-shusei, Tokyo, Japan) and 0.45 ml of low-melting-point agarose. The lung was then placed in ice-cold JMEM for 2 min to harden the agarose and incubated in 2 ml of the dispase solution for 20 min at room temperature. The lung was teased from the airways and minced in DMEM supplemented with penicillin (100 units/ml), streptomycin (100 μg/ml) and amphotericin B (0.75 μg/ml). The minced lung tissues were filtered successively through 133-, 42-, and 22-μm nylon mesh, and centrifuged at 130 g for 8 min. The resulting pellets were dispersed with DMEM supplemented with 10 % FCS and then plated on a petri dish for 4 h to remove mesenchymal cells and the floating cells (non adherent cells) were plated on fibronectin (Chemiconez-International, Temecula, CA, USA)-coated circular glass coverslips placed in the plastic dishes at density of 20000/dish. The viability of the isolated cells was checked by trypan-blue exclusion and was found to be greater than 98 %. Cells were maintained in DMEM supplemented with 10 % fetal calf serum at 37°C in 5 % CO_2 _and 95 % air. On the day 7 of culture, cells were immunocytochemically characterized using antibodies against pan-cytokeratin (which recognize cytokelatin 1–8, 10, 14–16, 19) (Nichirei Bioscience, Tokyo Japan) and vimentin (Dako, Carpinteria, CA, USA) as respective markers for epithelial cell and fibroblast. The majority of the cultured cells were epithelial cells with a uniform cobblestone pattern [21]. The contamination of fibroblasts was below 10 %. Macrophages or lymphocytes were not detected on the immnocytochemical analyses using antibodies against respective markers (Mac1 and CD45). Such cultures were subjected to experiments.

### Cell culture of A549 cells

A549 cells (human lung carcinoma-derived alveolar epithelial cell line) were cultured in DMEM supplemented with penicillin (100 units/ml), streptomycin (100 μg/ml), amphotericin B (0.75 μg/ml) and 10 % FCS at 37°C in 5 % CO_2 _and 95 % air. A549 cells were grown to 90 % confluence in 10 cm plastic culture dishes and harvested by exposure to 0.05 % trypsin and 0.02 % EDTA followed by centrifugation at 90 g for 1 min. The resulting pellets were resuspended in DMEM solution with the supplements and plated on the culture dishes for Western blotting analyses. After incubation at 37°C for 3 days to achieve 70–90% confluent monolayer, the cells were incubated in DMEM with or without 1 % FCS depending on experimental protocol.

### Immunocytochemistry in primary cultured alveolar epithelial cells and A549 cells

Primary cultured alveolar epithelial cells grown on coverslips glass incubated with DMEM containing 1 % FCS were stimulated with or without thrombin, AYPGKF-NH_2 _and TGF-β for 72 h at 37°C. The cells were fixed for 5 min with 1 % paraformaldehyde in a phosphate-buffered solution (PBS) containing 136.9 mM NaCl, 2.1 mM KCl, 3.2 mM Na_2_HPO_4 _and 1.5 mM KH_2_PO_4_, pH 7.4, and were washed twice with PBS. Fixed cells were permeated with 1 % Triton X-100 for 5 min, and were washed twice with PBS solution. The cells were incubated with monoclonal antibody against E-cadherin in PBS containing 2 mg/ml bovine serum albumin (BSA) for 2 h at room temperature. The cells were then incubated with FITC-conjugated monoclonal antibody against α-SMA and rhodamin red-conjugated goat anti-rabbit IgG and for 1 h at room temperature. On the other hand, after incubation with E-cadherin, A549 cells were incubated with monoclonal antibody against α-SMA (Nichirei Bioscience, Tokyo Japan) for 1 h at room temperature. Then, the cells were incubated with FITC-conjugated goat anti-mouse IgG and rhodamin red-conjugated goat anti-rabbit IgG and for 1 h at room temperature. Coverslips with the cells were mounted on a slide and visualized using confocal laser microscopy system (LSM 510-META; Carl Zeiss, Iena, Germany) with a water immersion objective lens for high resolution; 8 frames per slide were captured. Aperture, gain and black level for image acquisition were maintained at a constant level.

### Prepareation of cell lysates

A549 cells were stimulated with test drugs in DMEM in the presence (for E-cadherin and α-SMA detection) or absence (for pEGFR and pSrc detection) of 1 % FCS for the indicated periods followed by harvest. The cells were lysed at 4°C in the two kinds of cold lysis buffer. Triton buffer containing 30 mM HEPES (pH 7.1), 100 mM NaCl, 1 mM EGTA, 20 mM NaF, 1 % Triton X-100, 1 mM phenylmethylsulphonylfluoride (PMSF) and 20 μl/ml protease inhibitor cocktail (Roche Diagnostics GmbH, Mannheim, Germany) were used for detection of E-cadherin and α-SMA. RIPA (radio immunoprecipitation assay) buffer (pH 7.4) containing 100 mM HEPES, 300 mM NaCl, 2 mM EDTA, 2 % Nonidet P40, 1 % sodium deoxycholate, 0.2 % sodium lauryl sulfate (SDS), 3 mM MgSO4, 200 U/ml aprotinin, 20 μg/ml leupeptin, 1 mM PMSF, 100 mM NaF, 10 mM orthovanadate and 20 μl/ml protease inhibitor cocktail were used for detection of EGFR or Src phosphorylation. Both lysates obtained with each method were centrifuged 9000 g for 5 min. The supernatants were mixed with a SDS-Laemmli sample buffer and boiled for 2 min. Protein concentrations of the samples were measured using the Bio-Rad protein assay (Bio-Rad Laboratories, Hercules, CA, USA).

### Western blot analysis

Equal amounts of samples (20 μg/lane) were separated on 7.5 % SDS-polyacrylamide gel electrophoresis and transferred to an immobilon P membrane (Millipore Corporation, Bedford, MA, USA) using an electroblotting apparatus. The membrane was blocked with 5 % non-fat milk or 1 % BSA (for phosphorylated antibody) in Tris-buffered saline (TBS; 50 mM Tris and 150 mM NaCl, pH 7.5) for 1 h at room temperature. The membrane was incubated with each primary antibody overnight at 4°C. The membrane was then washed and incubated for with peroxidase-conjugated goat anti-rabbit or anti-mouse immunogloblin for 1 h at room temperature. The membrane was visualized by the enhanced chemiluminescence method. To confirm equal loading, the same membranes were stripped by incubating in a solution containing 100 mM 2-mercaptoethanol, 2 % SDS and 62.5 mM Tris-HCl (pH 6.8) for 1 h at 50°C, then blotted with the anti-GAPDH, anti-Src or anti-EGFR antibody.

The signal intensities were measured with a densitometer (DMU-33C; Advantec Digital Densitol, Tokyo, Japan).

### Statistical analysis

Statistical analyses were performed by ANOVA and Bonferroni's test. When only two groups were compared, Student's t-test was used for statistical analysis. The differences between mean values with P values less than 0.05 were considered significant.

## Results

### Morphological changes induced by PAR_4 _agonists

**Figure 1:**
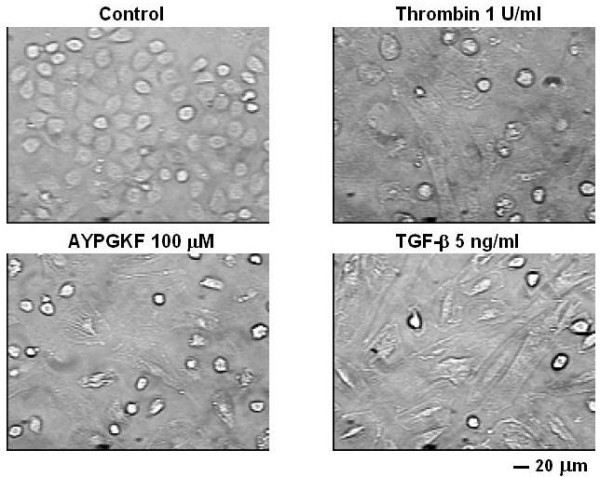
**Typical phase contrast images of primary cultured mouse alveolar epithelial cells**. Epithelial cells were treated for 72 h without agonist (control) or with thrombin (1 U/ml), PAR_4 _agonist peptide (AYPGKF-NH_2_, 100 μM) or TGF-β (5 ng/ml).

Figure 1 shows the typical morphological images of primary cultured alveolar epithelial cells assessed by phase contrast microscopy. Control cells formed monolayer in which individual cells displayed typical cobblestone-like shape and were tightly attached to each other. In contrast, after exposure to thrombin (1 U/ml) or a PAR_4 _agonist peptide (AYPGKF-NH_2_, 100 μM) for 72 h, many cells showed an elongated shape and lost cell-cell contact. When the cells were treated with TGF-β (5 ng/ml), a potent EMT inducer [8,10,22], such a morphological change was obvious, showing an extremely elongated shape like fibroblasts. PAR_4 _stimulation of alveolar epithelial cells thus appeared to induce phenotypic changes to mesenchymal-like cells.

### Changes in cell structure induced by PAR_4 _stimulation

**Figure 2:**
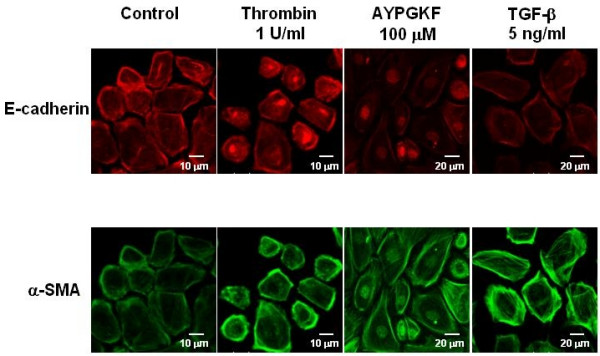
**Phenotypic changes in primary cultured alveolar epithelial cells stimulated with PAR_4 _agonists or TGF-β**. Immunofluorescence images for a specific marker for epithelial cell (E-cadherin; rhodamine red, upper panel) or myofibroblast (α-SMA; FITC green, lower panel) captured with confocal lasar microscopy. Cells were treated with or without (control) various agonists (thrombin, AYPGKF-NH_2 _or TGF-β) for 72 h, and stained for E-cadherin or α-SMA using specific antibodies as described in Method section.

**Figure 3:**
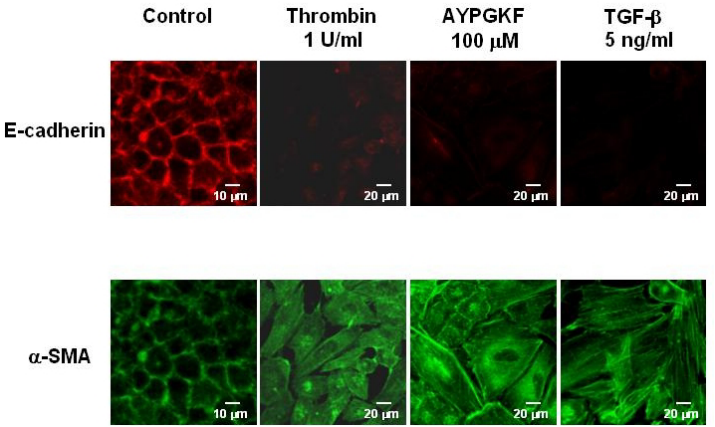
**Phenotypic changes in A549 cells stimulated with PAR_4 _agonists or TGF-β**. Immunofluorescence images for a specific marker for epithelial cell (E-cadherin; rhodamine red, upper panel) or myofibroblast (α-SMA; FITC green, lower panel) captured with confocal lasar microscopy. Cells were treated with or without (control) various agonists (thrombin, AYPGKF-NH_2 _or TGF-β) for 72 h, and stained for E-cadherin or α-SMA using specific antibodies as described in Method section.

To address whether PAR_4 _stimulation induces EMT in the primary cultured alveolar epithelial cells, changes in expression pattern of epithelial or myofibroblast marker was immunocytochemically analyzed. Figure 2 shows a representative immunofluorescence image of expression of E-cadherin or α-SMA, a typical marker of epithelial cell or myofibroblast, respectively. After 72 h exposure of the cells to either of PAR_4 _agonists, E-cadherin expression was significantly decreased concomitantly with reduced cell-cell contact and loss of epithelial morphology. In contrast, strong expression of α-SMA-positive microfilaments became clearly observed in the cytoplasmic peripheral area by the same treatments as compared with non-evident expression in control without stimulation. TGF-β(5 ng/ml, 72 h) used as a typical EMT inducer showed a marked decrease or increase in expression of E-cadherin or α-SMA, respectively. Such EMT responses were also observed in alveolar epithelial cell line (A549 cells) stimulated with PAR_4 _agonists and TGF-β(5 ng/ml) for 72 h (Fig. 3). In the next experiment, such immunocytochemical changes in E-cadherin and α-SMA expression was substantiated by Western blot analyses using A549 cells.

**Figure 4:**
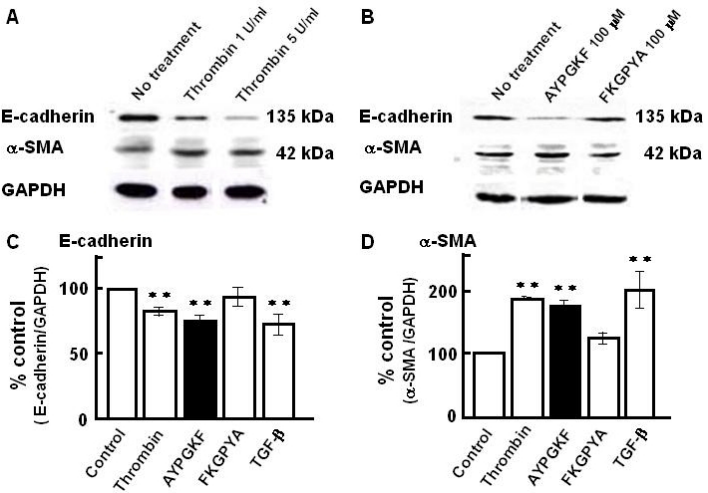
**Western blot analyses of E-cadherin and α-SMA in A549 epithelial cells stimulated with PAR_4 _agonists and TGF-β**. (A, B) Representative Western blots of E-cadherin and α-SMA in A549 cells treated for 96 h with thrombin (A; 1 or 5 U/ml) or synthetic peptides (B; AYPGKF-NH_2 _or FKGPYA-NH_2_; 100 μM). (C, D) Summarized densitometric data. Effects of PAR_4 _agonists (thrombin; 1 U/ml and AYPGKF-NH_2_; 100 μM), FKGPYA-NH_2 _(100 μM) or TGF-β (5 ng/ml) on the expressions of E-cadherin (C) and α-SMA (D). Results are based on densitometric analyses of the ratio of each marker protein to GAPDH (as internal standard). Each bar represents the mean ± S.E.M. from 4–12 preparations. **P < 0.01 compared with the control.

As shown in Figure 4A, treatment of A549 cells with thrombin (1 and 5 U/ml, 96 h) decreased the amount of immunoreactive E-cadherin (molecular mass, 135 kD), and increased α-SMA (molecular mass, 42 kD), compared with time-matched control (no treatment). A similar response to AYPGKF-NH_2 _(100 μM, 96 h), but not to a FKGPYA-NH_2 _(PAR_4_-inactive reverse sequence peptide, 100 μM), was also observed, suggesting the response to agonist peptide due to specific PAR_4 _stimulation (Figure 4B). Figure 4C and 4D show the pooled data obtained from 4–12 preparations. PAR_4_-mediated changes in expression level of each marker protein (decreases in E-cadherin or increases in α-SMA) were quantitated by the densitometric analyses. Reverse sequence peptide (FKGPYA-NH_2_, inactive form) at the same concentration (100 μM) did not show significant changes in the expressions of both marker proteins.

Thrombin-induced changes in each marker protein were not significantly affected by the PAR_1_-selective antagonist (SCH-79797, 300 nM) (E-cadherin; 78.3 ± 3.2 % or 75.6 ± 4.0 % of untreated group, α-SMA; 211.4 ± 23.6 % or 240.4 ± 45.6 % of untreated group, in thrombin-stimulated cells without or with inhibitor, respectively, mean ± S.E.M., n = 5), suggesting that the possibility of the participation of PAR_1 _in the thrombin action is unlikely. A potent EMT inducer, TGF-β (5 ng/ml) showed changes similar to those with PAR_4 _agonists.

In accordance with our observation, such a TGF-β-induced EMT in A549 cells was also detected by previous report [22,23]. Thus, PAR_4 _stimulation induced EMT in alveolar epithelial cells.

### Effects of inhibitors of EGF receptor kinase and Src kinase on PAR_4_-mediated EMT

**Figure 5:**
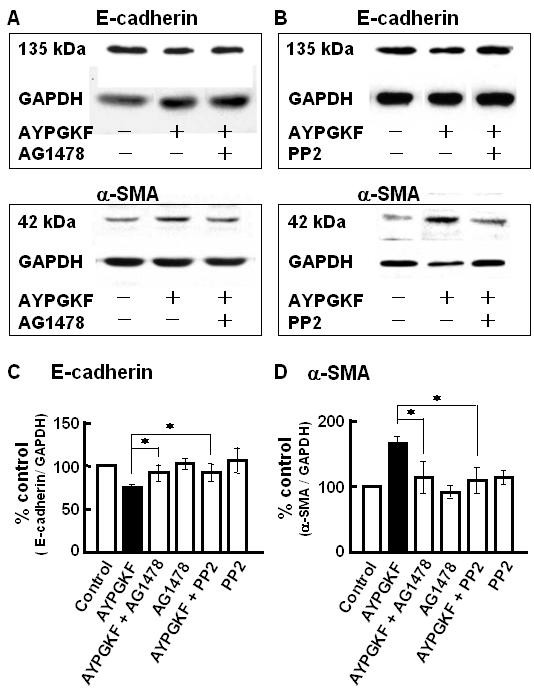
**Effects of inhibitors of EGFR tyrosine kinase and Src tyrosine kinase on PAR_4 _agonist-induced changes in EMT-related marker protein expression in A549 cells**. A549 cells were pretreatd with or without 30 nM AG1478 or 300 nM PP2 for 30 min, and then stimulated with AYPGKF-NH_2 _(100 μM) for 96 h followed by immunoblotting with specific antibodies for E-cadherin, α-SMA. (A, B) Representative Western blots for the effect of AG1478 (A) or PP2 (B) on the expression of E-cadherin and α-SMA. (C, D) Summarized densitometric data. Each bar represents the mean ± S.E.M. for 4–12 preparations. *P < 0.05 compared with AYPGKF-NH_2 _alone.

PARs reportedly transactivates, i.e., phosphorylates, EGFR in a variety of cell systems such as gastric cancer cells and cardiac myocytes [18,24], and phosphorylated EGFR triggers EMT in human ovarian surface epithelium [25]. Therefore, we examined whether EGFR is involved in the PAR_4_-mediated EMT, using an EGFR kinase inhibitor, AG1478 (Ki at 3 nM). As shown in Figure 5A (summarized in C and D), AG1478 inhibited both of the PAR_4 _stimulation-induced changes in the EMT parameters (E- cadherin and α-SMA), suggesting that EGFR signaling is involved in the PAR_4_-mediated EMT. Since EGFR transactivation is reportedly promoted by Src (18,24), the effects of PP2 (300 nM, a Src family tyrosine kinase inhibitor) were also examined. As shown in Figure 5B (summarized in C and D), PP2 inhibited the PAR_4_-mediated changes in E-cadherin and α-SMA expression, suggesting the involvement of Src signaling in the PAR4-mediated EMT.

### PAR_4 _stimulation-induced phosphorylation of EGF receptor and Src

**Figure 6:**
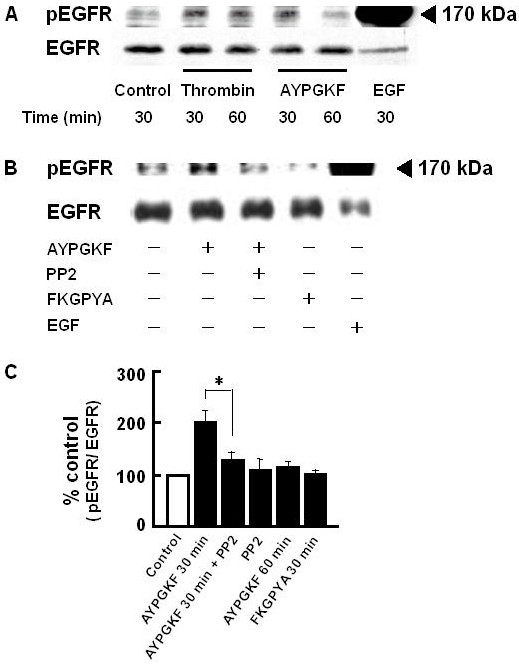
**PAR_4 _agonist-induced EGFR phosphorylation in A549 cells**. A549 cells were pretreated with or without 300 nM PP2 for 30 min, and then stimulated with AYPGKF-NH_2 _(100 μM) for 30–60 min followed by immunoblotting with specific antibodies for the Y1173 phosphorylated EGFR and total EGFR protein. Control; without drug treatment. (A) Representative time course of EGFR phosphorylation induced by thrombin (1 U/ml) or AYPGKF-NH_2 _(100 μM). As a positive control, cells were treated with EGF (10 ng/ml) for 30 min. (B) Representative Western blot after treatment with or without (control) various agents (AYPGKF-NH_2_, AYPGKF-NH_2_+PP2, 100 μM FKGPYA-NH_2 _or EGF) for 30 min. (C) Summarized densitometric data. Protein concentrations used for separation was 20 μg/lane (thrombin or AYPGKF-NH_2 _or FKGPYA-NH_2 _treated sample) or 10 μg/lane (EGF treated sample). Each bar represents the mean ± S.E.M. for 4–6 preparations. *P < 0.05 compared with AYPGKF-NH_2 _alone.

PAR_4 _stimulation induced phosphorylation of EGFR was examined. In A549 cells stimulated with thrombin (1 U/ml) or AYPGKF-NH_2 _(100 μM) for 30 or 60 min, tyrosine1173 phosphorylated EGFR (pEGFR) level was obviously elevated (Figure 6A and 6C). Such induction of pEGFR by PAR_4 _stimulation occurred as early as 30 min after the agonist application. EGF (10 ng/ml) as a positive control induced a marked increase in EGFR phosphorylation. Further, this PAR_4 _stimulation-induced increase in pEGFR level was significantly inhibited by PP2 (Figure 6B and 6C), suggesting that Src may be an upstream signaling pathway for the PAR_4 _stimulation-induced EGFR transactivation. EGFR phosphorylation was not observed by the stimulation with FKGPYA-NH_2 _(100 μM) for 30 min.

**Figure 7:**
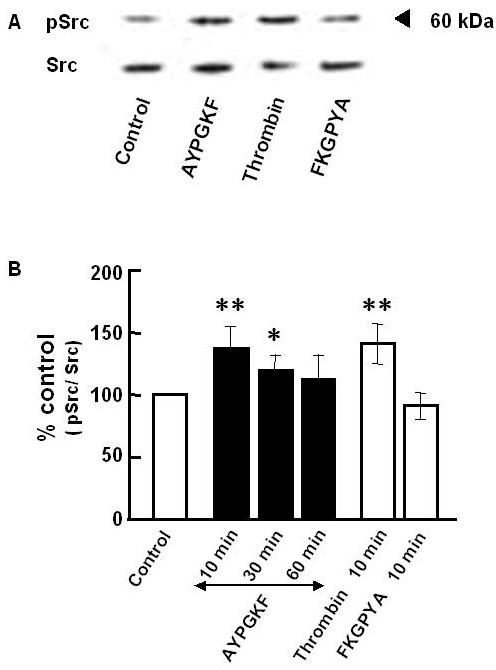
**Western blot analyses of Src phosphorylation in A549 cells**. A549 cells were treated without (control) or with AYPGKF-NH_2 _(100 μM) for 10, 30 or 60 min or with thrombin (1 U/ml, 10 min) or with FKGPYA-NH_2 _(100 μM, 10 min), and then subjected to immunoblotting with specific antibodies for the Y418 phosphorylated Src and total Src protein. (A) Representative Western blot. Stimulation time was 10 min. (B) Summarized densitometric data. Each bar represents the mean ± S.E.M. for 4–6 preparations. **P < 0.01, *P < 0.05 compared with the control.

To test whether PAR_4 _stimulation activates Src, the level of Tyr416 phosphorylated Src (pSrc, activated form of Src) was measured. As shown in Figure 7, stimulation of A549 cells with PAR_4_-agonist peptide (AYPGKF-NH_2_, but not FKGPYA-NH_2_, caused a rapid phosphorylation of Src. Thrombin (1 U/ml, 10 min) also induced an increase in pSrc level. PAR_4 _stimulation thus activated Src followed by EGFR transactivation to produce EMT in lung alveolar epithelial cells.

### The effects of EGFR kinase and Src kinase inhibitors on PAR_4 _stimulation-induced phenotypic changes in primary cultured alveolar epithelial cells

**Figure 8:**
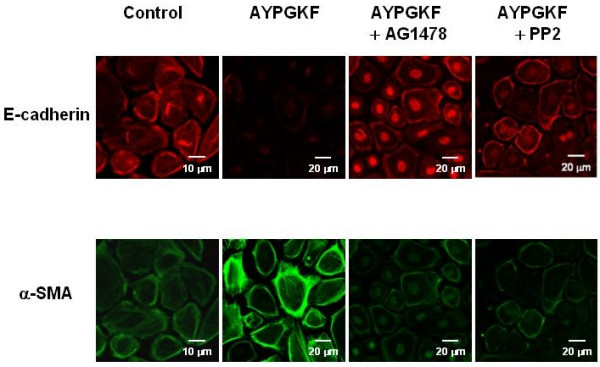
**Effects of AG1478 and PP2 on PAR_4 _agonist induced phenotypic changes in primary cultured alveolar epithelial cells**. Immunofluorescence images for a specific marker for epithelial cell (E-cadherin; rhodamine red, upper panel) or myofibroblast (α-SMA; FITC green, lower panel) captured with confocal lasar microscopy. Cells were treated with or without (control) AYPGKF-NH_2 _(100 μM) for 72 h in the presence or absence of each inhibitor (30 nM AG1478 or 300 nM PP2), and stained using each specific antibody as described in Method section.

Finally, we immunocytochemically analysed the expression of E-cadherin and α-SMA in the primary cultured alveolar epithelial cells exposed to AYPGKF-NH_2 _(100 μM, 72 h) in the presence of EGFR kinase inhibitor (AG1478, 30 nM) or Src family tyrosine kinase inhibitor (PP2, 300 nM). As shown by representative fluorescence imaging data in Figure 8, pretreatment with each inhibitor for 30 min obviously reduced the PAR_4 _agonist peptide-induced EMT parameter responses (loss of cell-cell contact, decrease in expression of E-cadherin and increase in expression of α-SMA), reinforcing the Western blot data. Each inhibitor alone unaffected cell morphology (data not shown).

## Discussion

In the present study, we showed that PAR_4 _stimulation of alveolar epithelial cells (primary cultured mouse epithelial cells and human A549 cell line) for 72–96 h resulted in the loss of a marker for epithelial cell (E-cadherin) and induction of a marker of myofibroblast (α-SMA). This is the first report demonstrating that PAR_4 _stimulation induces EMT in the primary cultured alveolar epithelial cells. Since these phenotypic changes were inhibited by inhibitors of EGFR kinase (AG1478) or Src family tyrosine kinase (PP2), the Src/EGFR-regulated mechanism is assumed to underlie the induction of EMT.

Molecular mechanisms of EMT induction are not clarified yet. A typical EMT inducer, TGF-β reportedly activate Smad 2 or Smad 3 signaling [10,12] or Rho/ROCK signaling [25] to induce EMT. Activated Smads translocate into the nucleus and facilitate transcription of target genes, for example α-SMA. Besides these signaling, the present findings showed new EMT-producing signaling pathway including activation of Src and EGFR.

Salient findings in this study were as follows; 1) PAR_4 _stimulation of alveolar epithelial cells induced morphological change to fibroblast-like cell shape, 2) changes in EMT parameters in response to PAR_4 _agonist (AYPGKF-NH_2_) were suppressed by inhibitors of EGFR (AG1478) and Src family (PP2) tyrosine kinases, 3) PAR_4 _stimulation resulted in elevation of pSrc and pEGFR levels, the latter being reduced in the presence of PP2. Based on these findings, PAR_4_-mediated EMT is considered to be produced through Src-mediated EGFR activation. Inhibitory effects of AG1478 or PP2 on PAR_4 _stimulation-induced changes in EMT parameters (Fig. 8) support our hypothesis. In line with this idea, EGF reportedly enhances TGF-β-induced EMT in human proximal tubular cells [27] and to induce EMT in human ovarian surface epithelium [25]. Further, treatment of A549 cells with EGF (10 ng/ml) for 96 h induced a phenotypic change as demonstrated by expression of marker proteins (E-cadherin; **84.5 ± 2.2 %, α-SMA; **305.5 ± 7.4% compared to respective untreated group, mean ± S.E.M. n = 3, **P < 0.05, the author's unpublished data). These informations reinforce the involvement of EGFR stimulation in EMT development. As downstream signaling to induce EMT after EGFR activation, enhanced activities of PI3 kinase, Akt, ERK and/or p38 MAPK that regulate transcription of various target proteins may be plausible candidates [25,28].

## Conclusion

**Figure 9:**
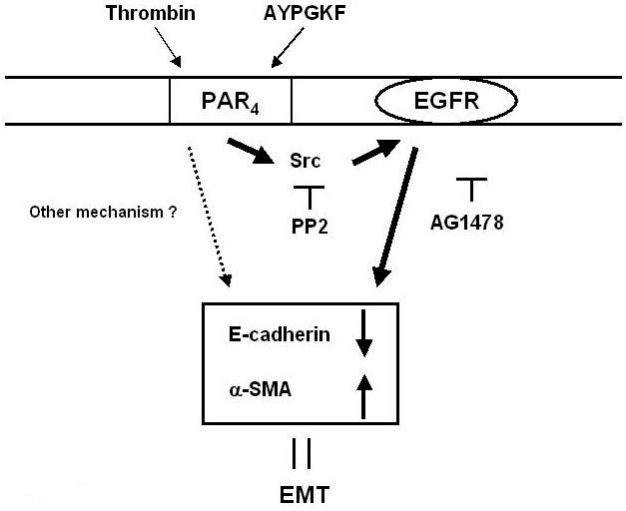
**A schematic illustration summarizing the mechanisms for PAR_4_-mediated EMT in alveolar epithelial cell**. Bold line: a new pathway proposed in this study. Dotted line: other mechanisms.

PAR_4 _stimulation of alveolar epithelial cells changed epithelial shape, and induced a decrease or an increase in expression of epithelial or myofibroblast marker, respectively, which is suggestive of EMT. Receptor-linked Src activation followed by EGFR transactivation was thought to be involved in this PAR_4_-mediated phenotypic changes in alveolar epithelial cells as summarized in Figure 9. Selective inhibition of PAR_4 _and/or this receptor-related signaling may yield a novel strategy to inhibit proteinase-mediated development of pulmonary fibrosis.

## Competing interests

The author(s) declare that they have no competing interests.

## Authors' contributions

SA and HO conceived of this study, and carried out acquisition, analysis and interpretation of data, and further prepared the manuscript.

YY helped Western blot experiment.

KK and HA participated in the synthesis of PAR_4_-related peptides.

SF and CI participated in coordination of data and helped to draft the manuscript.

All authors read and approved the final manuscript.
